# *PNPLA3* rs738409 Genetic Variant Inversely Correlates with Platelet Count, Thereby Affecting the Performance of Noninvasive Scores of Hepatic Fibrosis

**DOI:** 10.3390/ijms242015046

**Published:** 2023-10-10

**Authors:** Marica Meroni, Paola Dongiovanni

**Affiliations:** Medicine and Metabolic Diseases, Fondazione IRCCS Cà Granda Ospedale Maggiore Policlinico, Via Francesco Sforza 35, 20122 Milan, Italy; maricameroni11@gmail.com

**Keywords:** MASLD, platelets, *PNPLA3*, genetics, diagnostic accuracy

## Abstract

Noninvasive tests (NITs) including platelets (PLTs) have been proposed to replace hepatic biopsy for the diagnosis of nonalcoholic fatty liver disease (NAFLD), or as more recently redefined, metabolic dysfunction-associated steatotic liver disease (MASLD). There has been reported an inverse correlation between PLTs and progressive MASLD, which is also affected by the patatin-like phospholipase domain-containing protein 3 (*PNPLA3)* rs738409 C>G mutation. However, the correlation between low PLTs and *PNPLA3* genotype has been poorly investigated. We stratified 1155 biopsy-proven MASLD patients according to *PNPLA3* genotype. The hepatic expression of genes involved in megakaryopoiesis was investigated in *n* = 167 bariatric patients by RNAseq. PLT count progressively decreased according to the number of *PNPLA3* at-risk alleles, irrespective of the presence of advanced fibrosis. The hepatic expression of genes involved in PLT biogenesis was associated with the *PNPLA3* GG genotype. Finally, the presence of the *PNPLA3* homozygosity flattened the accuracy of fibrosis-4 (FIB-4) in discriminating histological fibrosis stages. The *PNPLA3* GG genotype may underpower the accuracy of NITs which include PLT count in identifying those patients with potentially reversible stages of fibrosis.

## 1. Introduction

Nonalcoholic fatty liver disease (NAFLD) is regarded as the most rapidly growing noncommunicable chronic disease of this century, affecting around 30% of the global population [1]. It consists of a wide spectrum of hepatic abnormalities ranging from steatosis to nonalcoholic steatohepatitis (NASH), which may progress to fibrosis and eventually to hepatocellular carcinoma (HCC) [2]. Recently, the novel nomenclature metabolic dysfunction-associated steatotic liver disease (MASLD) has been proposed to redefine NAFLD, thus being more inclusive of cardiometabolic risk factors [3]. As a complex disorder, MASLD has a convoluted pathogenesis in which both environmental and genetic factors coexist [4].

Specifically, 50–70% of the individual susceptibility to develop the disease as well as its phenotypic variability are attributable to inherited risk factors. Since the first genome-wide association studies (GWAS) [5], the current knowledge regarding the genetic predisposition to MASLD supports the primary role of the rs738409 (p.I148M, C>G) variant in patatin-like phospholipase domain-containing protein 3 (*PNPLA3*) gene. In particular, it has been pointed out as the most robust genetic predictor of severe MASLD, possibly explaining the interindividual and ethnicity-related variability in hepatic fat content [5]. The size effect of this inherited variant is so overwhelming that it is still considered the strongest ever reported for a common variant, projecting the proportion of the total variance attributed to this polymorphism at 5.3% [6].

The *PNPLA3* gene codifies a membrane lipase, exerting its function in hydrolyzing the lysophosphatidic acid to phosphatidic acid. It is localized in the endoplasmic reticulum (ER) and at the lipid droplet (LD) surface in hepatocytes, adipocytes and in hepatic stellate cells (HSCs) [4]. The p.I148M mutation is associated with reduced PNPLA3 enzymatic activity, abolished proteasomal degradation, global metabolic perturbations, lower lipid mobilization and severe liver damage [4].

To date, although several novel noninvasive tests (NITs) and biomarkers have gained trajectory in the clinical guidelines, data regarding non-intrusive MASLD diagnosis are still inconclusive. Some of these NITs, among them fibrosis-4 (FIB-4) and AST-to-platelet ratio index (APRI), include the evaluation of transaminase levels as markers of liver damage along with the platelets (PLTs).

PLTs participate in different pathological processes guiding the progression of the disease, including inflammation and fibrogenesis. Differently from simple steatosis, PLT number and activation is exacerbated during NASH, further aggravating the inflammatory status [7]. In addition, when PLTs are recruited to the liver, they can aggregate and release pro-fibrogenic factors (cytokines, chemokines, platelet-derived growth factor (PDGF) and transforming growth factor-beta (TGFβ)) to foster wound healing and regeneration on one hand and activate HSCs on the other. The excessive deposition of collagen fibers by HSCs alters liver zonation by precluding the influx of oxygen and metabolites to hepatic cells. In addition, a hypoxic microenvironment favors fibrosis worsening, cirrhosis, HCC development and angiogenesis. Severe liver injuries, portal hypertension and cirrhosis are the best-known causes of thrombocytopenia, due to reduced hepatic thrombopoietin secretion and increased PLT destruction by enlarged spleens [8,9]. Notably, it has been estimated that the 54–87% of variability in PLT number is shaped by genetic traits [10]. Nonetheless, the genetic variants shared by PLT count and liver damage are not well established.

In 2021, Pirola and colleagues aimed at identifying for the first time the potential molecular mediators that may possibly link MASLD to PLTs [11]. These authors demonstrated that the most significantly associated loci to the genetic modulation of PLT count are those implicated in metabolic-related pathways [11]. Accordingly, the *PNPLA3* rs738409 variant has been identified as a modifier of the PLT count by exome-chip meta-analysis in 157,293 individuals [12], supporting the concept that genetic variants associated with PLT count are predictive of human diseases. However, the impact of *PNPLA3* on PLTs is still largely unexplored.

Therefore, in the attempt to identify the contribution of *PNPLA3* variant to PLT count, we stratified 1155 biopsy-proven MASLD patients according to the *PNPLA3* rs738409 (p.I148M, C>G) variant (*n* = 484 CC (41.9%), *n* = 485 CG (42%) and *n* = 186 GG (16.1%)) (Table 1 and Table S1). As we expected, patients carrying the *PNPLA3* rs738409 variation in heterozygosity or homozygosity displayed a more severe steatosis, an elevated grade of lobular inflammation and ballooning and raised stages of fibrosis compared to non-carriers, thus confirming its well-established role in inducing advanced liver damage.

## 2. Results and Discussion

**Figure 1:**
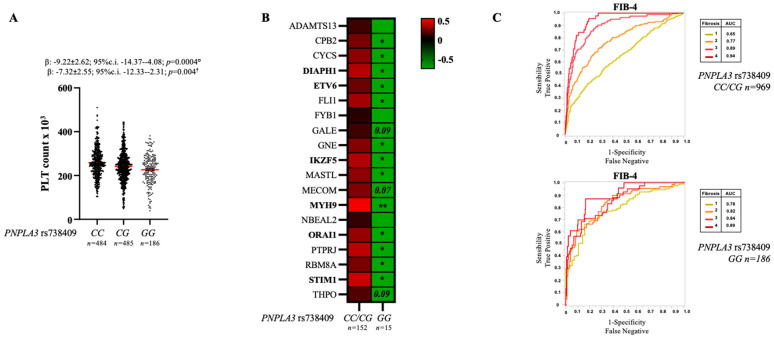
Impact of the *PNPLA3* genotype on PLT count and its role in influencing noninvasive tests in predicting fibrosis stage. PLTs distribution in 1155 MASLD patients, stratified according to the presence of the *PNPLA3* rs738409 (p.I148M, C>G) variant (*n* = 484 CC (41.9%); *n* = 485 CG (42%) and *n* = 186 GG (16.1%)). The estimates (β) were obtained from ordinal logistic regression analysis adjusted for gender, age, body mass index (BMI), type 2 diabetes (T2D) by using and additive (Model 1 °) or recessive model (Model 2 ^†^) (**A**). The heatmap represents the expression of genes involved in PLT biogenesis and clearance in hepatic samples from *n* = 167 MASLD patients stratified according to the presence of *PNPLA3* homozygosity (*n* = 152 CC/CG and *n* = 15 GG). The genes highlighted in bold remained significantly associated with *PNPLA3* GG genotype in multivariate analysis adjusted for gender, age, BMI, T2D by using a recessive model (* *p* < 0.05; ** *p* < 0.01) (**B**). ROC curves describe the accuracy of the noninvasive score, FIB-4 in discriminating the histological stage of fibrosis in 1155 MASLD patients stratified according to the presence of PNPLA3 homozygosity (*n* = 969 CC/CG and *n* = 186 GG). Area under the curves (AUC) is reported in the graphs (**C**). ADAMTS13: ADAM Metallopeptidase With Thrombospondin Type 1 Motif 13, CPB2: Carboxypeptidase B2; CYCS: Cytochrome C, Somatic; DIAPH1: Diaphanous Related Formin 1; ETV6: ETS Variant Transcription Factor 6; FLI1: Fli-1 Proto-Oncogene; ETS: Transcription Factor; FYB1: FYN Binding Protein 1; GALE: UDP-Galactose-4-Epimerase; GNE: Glucosamine (UDP-N-Acetyl)-2-Epimerase/N-Acetylmannosamine Kinase; IKZF5: IKAROS Family Zinc Finger 5; MASTL: Microtubule Associated Serine/Threonine Kinase Like; MECOM: MDS1 And EVI1 Complex Locus; MYH9: Myosin Heavy Chain 9; NBEAL2: Neurobeachin Like 2; ORAI1: ORAI Calcium Release-Activated Calcium Modulator 1; PTPRJ: Protein Tyrosine Phosphatase Receptor Type J; RBM8A: RNA Binding Motif Protein 8A; STIM1: Stromal Interaction Molecule 1; THPO: Thrombopoietin.

In our cohort, we found that PLT count progressively decreased according to the number of *PNPLA3* at-risk alleles (228.9 ± 74.2 PLTs in GG vs. 242.7 ± 71.8 in CG and 256.1 ± 68.9 in CC) and more so in homozygous patients (228.9 ± 74.2 PLTs in GG vs. 250 ± 70 in CC/CG). PLTs decreased almost 5% in CG patients and 10% in GG patients, compared to those carrying the wild-type genotype (Table 1 and Figure 1A). Moreover, at multivariate analysis adjusted for gender, age, body mass index (BMI), type 2 diabetes (T2D), the *PNPLA3* at-risk G allele was associated with reduced PLTs (β: −9.22 ± 2.62; 95%c.i. −14.37–4.08; *p* = 0.0004; additive model; β: −7.32 ± 2.55; 95%c.i. −12.33–2.31; *p* = 0.004; recessive model).

To exclude that this association may be driven by the more severe liver damage observed in *PNPLA3* carriers, we further adjusted the analysis for the presence of advanced fibrosis (fibrosis stage > 2). We observed that the *PNPLA3* genotype was associated with PLT count, independently of fibrosis stage > 2 (β: −6.84 ± 2.58; 95%c.i. −11.90–1.77; *p* = 0.008; additive model; β: −4.49 ± 2.53; 95%c.i. −9.45–0.46; *p* = 0.06; recessive model). Notably, we did not find any association between the protective *PNPLA3* rs2294918 G>A (p.E434K) variant and PLT levels (Figure S1).

In line with this evidence, in the UK Biobank cohort (UKBBC) the most significantly correlated trait with *PNPLA3* rs738409 was PLT count (β: −1.54 vs. C genotype; *p* = 2.9 × 10^−45^). Then, we found hemoglobin concentration (β: 0.02 vs. C genotype; *p*= 9.09 × 10^−35^), platelet crit (β: −0.001 vs. C genotype; *p* = 3.62 × 10^−29^), hematocrit percentage (β: 0.06 vs. C genotype; *p* = 2.94 × 10^−25^) and K70-K77 diseases of liver (β: 0.003 vs. C genotype; *p* = 3.38 × 10^−25^) (Table S2). Conversely, the association with mean platelet volume, a marker of increased cardiovascular risk in patients with NASH [8,13] was positive (β: 0.01 vs. G genotype; *p* = 9.53 × 10^−18^). Hence, the strength of the association of *PNPLA3* with reduced PLT levels exceeded that of association with liver diseases, in UKBBC. However, only a few reports previously highlighted this inverse correlation between the *PNPLA3* genotype and PLTs and mainly in small cohort of patients with viral hepatitis C [14,15,16].

The likely mechanism behind this association is still unknown, since there are no currently established relationships between *PNPLA3* and PLTs. Indeed, the mRNA and protein expressions of PNPLA3 in megakaryocytes and PLTs are almost absent (www.proteinatlas.org/ENSG00000100344-PNPLA3, accessed on 1 August 2023). Hence, we could only hypothesize possible explanations, encompassing low production of PLTs from bone marrow or excessive oxidative stress, causing mitochondrial dysfunction, cytochrome c release and caspase-3 activation, leading to apoptosis of subgroups of PLTs in MASLD patients carrying the *PNPLA3* variant, similarly to what has been described by Tang et al., in diabetic patients [17]. Likewise, we have to take into account that during the embryogenesis, the fetal liver is the privileged organ for megakaryocyte progenitor differentiation from stem cell niche and therefore we could speculate that the presence of the variant may affect megakaryopoiesis during the development, thereby altering the number of PLT progenitors for the individuals’ entire life [18]. In addition, the liver is the main organ responsible for the production of thrombopoietin, which is the primary regulator of PLT synthesis for the lifetime [19].

Therefore, we could speculate that genetic variants such as that in the *PNPLA3* gene that strongly impact on liver function may possibly affect also PLT biogenesis. In this regard, we decided to investigate the hepatic expression of thrombopoietin and other genes involved in megakaryopoiesis and in which loss-of-function mutations are causative of hereditary thrombocytopenia, in a cohort of MASLD bariatric patients from whom RNAseq data were available (transcriptomic cohort; *n* = 167) (Table S3). We observed that, the hepatic expression of thrombopoietin and of other genes which participate in PLT biogenesis, most of them localized in the liver or essential for the transition of hematopoietic activity from fetal liver to bone marrow, were overall downregulated in patients homozygous for the *PNPLA3* variant (Figure 1B). At multivariate analysis adjusted for gender, age, BMI, T2D and presence of NASH, the hepatic expression of *DIAPH1* and *MYH9*, which are involved in cytoskeleton remodeling and pro-platelet formation as well as that of *ORAI1* and *STIM1*, which form a Ca^2+^ selective ion channel participating to mast cell degranulation, remained significantly reduced in *PNPLA3* homozygous patients. Similarly, *ETV6* and *IKZF5* genes, which encode transcription factors involved in megakaryopoiesis, were associated with the *PNPLA3* GG genotype independently of the confounding factors mentioned above. All these genes have been found to be positively correlated with *PNPLA3* gene expression in our cohort (Table S4). Therefore, the down-modulation of these genes was independent of severe liver disease, further supporting a genetically driven reduction of PLT count.

In the era in which the efforts are focused on the discovery of novel NITs to diagnose NASH and predict disease progression, these observations gain particular interest. Indeed, it should be of primary importance to take into account that genetic background may possibly affect biochemical factors included in these scores, partially altering their efficacy. Accordingly, Liu WY et al. reported a suboptimal performance of FIB-4 in patients carrying the *PNPLA3* genotype compared to non-carriers [20]. We observed similar findings in our cohort of MASLD patients, in whom the presence of the *PNPLA3* homozygosity flattened the diagnostic accuracy of FIB-4 in discriminating histological fibrosis stages (Figure 1C). Superimposable results have been also obtained by considering APRI and Forns index in patients carrying the GG *PNPLA3* genetic background (Figure S2A,B).

Hence, we can conclude that the homozygosity for *PNPLA3* genotype may underpower the accuracy of NITs which include PLT count in identifying those patients with potentially reversible stages of fibrosis.

Unfortunately, *PNPLA3* genotyping is not performed routinely in all diagnostic centers. Thus, the simultaneous assessment of different NITs combined with imaging techniques remains the only way to noninvasively detect MASLD and its progressive forms, although their clinical utility may be affected by genetics.

## 3. Material and Methods

### 3.1. Liver Biopsy Cohort

The overall cohort consisted of 1155 patients with histological diagnosis of MASLD (liver biopsy cohort), as previously described [21]. Briefly, 1155 adult individuals were consecutively enrolled at the metabolic liver diseases outpatient service (*n* = 571) and the bariatric surgery center (*n* = 584) at Fondazione IRCCS Ca’ Granda Ospedale Maggiore Policlinico (Milan, Italy). Inclusion criteria were availability of liver biopsy performed for suspected NASH or severe obesity, availability of DNA samples and clinical data. Individuals with excessive alcohol intake (men, >30 g/day; women, >20 g/day), viral and autoimmune hepatitis, hereditary hemochromatosis and alpha1-antitrypsin deficiency or other causes of liver disease were excluded. Informed written consent was obtained from each patient and the study protocol was approved by the Ethical Committees of the Fondazione IRCCS Ca’ Granda, Milan (811_2021; 401_2019), and conformed to the ethical guidelines of the 1975 Declaration of Helsinki. The clinical characteristics of patients evaluated in the study are listed in Table 1 and Table S1.

### 3.2. Histological Evaluation

Steatosis was graded according to the percentage of affected hepatocytes as 0: 0–4%, 1: 5–32%, 2: 33–65%, and 3: 66–100%. Disease activity was assessed according to the MASLD activity score (NAS) with systematic evaluation of hepatocellular ballooning and necroinflammation; fibrosis was also staged according to the recommendations of the MASLD Clinical Research Network. The scoring of liver biopsies was performed by independent pathologists unaware of patients’ status and genotype. NASH was diagnosed when (a) steatosis, (b) lobular inflammation and (c) ballooning were concomitantly present.

The noninvasive assessment of fibrosis was performed by using Fibrosis-4 (FIB-4), AST-to-platelet ratio index (APRI) and Forns index. FIB-4 was calculated according to the following formula “age × AST (IU/l)/platelet count (×109/l) × √ ALT (IU/l)”, whereas APRI as “(AST/upper limit of the normal AST range) × 100/platelet count” and Forns index = 7.811 − 3.131 × ln (PLT count) + 0.781 × ln (GGT) + 3.467 × ln (age) − 0.014 × cholesterol.

### 3.3. Genotyping

The overall cohort was genotyped for the rs738409 C>G (PNPLA3 p.I148M) variants using TaqMan 5′-nuclease assays in duplicate (QuantStudio 3, Thermo Fisher, Waltham, MA, USA), as previously described [21]. The success rate of genotyping was >99%.

### 3.4. UK Biobank Cohort

The association between the rs738409 C>G (PNPLA3 p.I148M) variant and phenotypes related to hematologic traits and liver disease were evaluated in the UK Biobank cohort (UKBBC). UKBBC is a prospective population-based study of approximately 500,000 individuals not selected for liver diseases and ethnicity, almost all aged 40–69 years, identified in 22 centers across the UK during 2006–2010. Freely available basic association data were downloaded from Neale Lab in August 2023 (http://geneatlas.roslin.ed.ac.uk, accessed on 1 August 2023) and *p*-values were corrected for multiple testing using the false discovery rate (FDR) method [22]. The associations between the *PNPLA3* rs738409 C>G variant, hematological parameters and liver-related outcomes in the UK Biobank cohort (UKBBC) are listed in Table S2.

### 3.5. Transcriptomic Analysis

RNA-seq was performed in a subset of 167 severely obese patients belonging to the liver biopsy cohort, of whom percutaneous liver biopsy was performed during bariatric surgery at Fondazione IRCCS Cà Granda, Ospedale Maggiore Policlinico [23]. The study conformed to the Declaration of Helsinki and was approved by the Institutional Review Board and Ethics Committee. All participants gave written informed consent. Clinical characteristics of the transcriptomic cohort are presented in Table S3. RNA-seq mapping descriptive statistics, the detailed protocol and data analysis approach have been previously described [22,23].

Briefly, total RNA was isolated from hepatic specimens using an RNeasy mini-kit (Qiagen, Hulsterweg, Germany). RNA quantification and quality control (QC) were determined by NanoDrop ND-1000 spectrophotometer (Thermo Fisher Scientific, Carlsbad, CA, USA) and Agilent 2100 Bioanalyzer (Thermo Fisher Scientific, Carlsbad, CA, USA), respectively. Samples with an RNA integrity number (RIN) ≥ 7 were used for library preparation.

RNA was sequenced in paired-end mode (read length 150 nt) using the Illumina HiSeq 4000 (Novogene, Hong Kong, China). The quality of the reads was determined using FastQC software (Babraham Bioinformatics, Cambridge, UK; https://www.bioinformatics.babraham.ac.uk/projects/fastqc, accessed on 1 August 2023) and low-quality sequences were trimmed using Cutadapt. Reads were aligned to the human reference genome (GRCh38/hg38) using STAR [5], with standard input parameters, and gene counts were produced using Subread featureCounts [6] using Genecode (v31) as gene annotation.

### 3.6. Statistical Analysis

Statistical analyses were performed using JMP 16.0 Pro (SAS, Cary, NC, USA), R statistical analysis version 3.3.2 (http://www.R-project.org/, accessed on 1 August 2023) and Prism (version 9, GraphPad Software Inc, San Diego, CA, USA), using one-way analysis of variance (ANOVA) or chi-square test where appropriate.

For descriptive statistics, continuous variables are shown as mean and standard deviation or median and interquartile range for highly skewed biological variables (i.e., AST, ALT, triglycerides (TGs)). Variables with skewed distributions were logarithmically transformed before analyses. Categorical variables are presented as number and proportion. All genetic analyses were performed under additive and recessive models.

Analyses were performed by fitting data to generalized linear regression models. Generalized linear models were fitted to examine continuous traits. Multinomial logistic regression models were fitted to examine binary traits (cirrhosis, HCC), and ordinal regression models were fitted for ordinal traits (components of the MASLD activity score: severity of steatosis, necroinflammation and hepatocellular ballooning, stage of fibrosis). When specified, confounding factors were included in the model. For gene expression analyses, differences between groups were calculated by one-way ANOVA, which was followed by post hoc *t*-tests adjusted for the number of comparisons when multiple groups were involved (Bonferroni correction or Benjamini-Hochberg false discovery rate (FDR) correction, where indicated). *p*-values < 0.05 (two-tailed) were considered statistically significant.

## Figures and Tables

**Table 1:** Demographic, anthropometric and clinical features of the liver biopsy cohort (*n* = 1155) stratified according to the *PNPLA3* rs738409 C>G genotype.

	CC (*n* = 484)	CG (*n* = 485)	GG (*n* = 186)	*p*-Value °	*p*-Value ^†^
Sex, M/F	222/262	236/249	88/98	0.68	0.99
Age, years	47.3 ± 12.6	47.9 ± 12.6	50.5 ± 13.4	**0.02**	**0.005**
BMI, kg/m^2^	35.8 ± 8.55	35.3 ± 8.87	33.2 ± 8.0	**0.001**	**0.0004**
IFG/T2D (0/1)	387/97	352/133	139/47	**0.03**	0.65
Total cholesterol, mmol/L	5.08 ± 1.05	5.08 ± 1.07	5.00 ± 1.13	0.87 *	0.91 *
LDL cholesterol, mmol/L	3.14 ± 0.96	3.17 ± 0.96	3.14 ± 0.97	0.21 *	0.53 *
HDL cholesterol, mmol/L	1.34 ± 0.38	1.26 ± 0.35	1.30 ± 0.36	<0.0001 *	0.07 *
Triglycerides, mmol/L	1.51 ± 0.84	1.68 ± 1.15	1.54 ± 0.86	0.07 *	0.50 *
ALT, IU/l	27 {19–47}	32 {20–56}	44 {28–69}	<0.0001 *	<0.0001 *
AST, IU/l	22 {18–32}	25 {18–37}	32 {24–48}	<0.0001 *	<0.0001 *
GGT, IU/l	37 {21–84}	39 {22–74}	35 {22–94}	0.51 *	0.95 *
Steatosis ≥ 2, yes (%)	203 (42)	238 (49)	119 (64)	<0.0001 *	<0.0001 *
Lobular inflammation ≥ 1, yes (%)	290 (60)	310 (64)	149 (80)	<0.0001 *	<0.0001 *
Ballooning ≥ 1, yes (%)	116 (24)	150 (31)	67 (36)	0.001 *	0.06 *
Fibrosis ≥ 2, yes (%)	77 (16)	107 (22)	71 (38)	<0.0001 *	<0.0001 *
PLT count × 10^3^	256.1 ± 68.9	242.7 ± 71.8	228.9 ± 74.2	0.0004 *	0.004 *
FIB-4	1.00 ± 0.84	1.26 ± 1.66	1.73 ± 2.40	0.0001 *	0.0005 *
APRI	0.25 ± 0.23	0.33 ± 0.51	0.46 ± 0.48	<0.0001 *	<0.0001 *
Forns index	4.22 ± 2.02	4.46 ± 1.93	4.97 ± 2.43	0.02 *	0.006 *

Values are reported as mean ± SD number (%) or median {IQR}, as appropriate. BMI: body mass index. IFG: impaired fasting glucose. T2D: type 2 diabetes. Characteristics of participants were compared across the rs738409 genotypes using generalized linear model (for continuous characteristics) or nominal logistic regression model (for categorical characteristics). *p* value < 0.05 are highlighted in bold. * Models were adjusted for gender, age, BMI, IFG/T2D, PNPLA3 p.I148M alleles. °Additive model, ^†^ recessive model.

## Data Availability

All datasets generated for this study are included in the article. Further inquiries can be directed to the corresponding author.

## Supplementary Materials

The following supporting information can be downloaded at: https://www.mdpi.com/article/10.3390/ijms242015046/s1.
