# Supplementary material for: PNPLA3 rs738409 Genetic Variant Inversely Correlates with Platelet Count, Thereby Affecting the Performance of Noninvasive Scores of Hepatic Fibrosis
Source: Int J Mol Sci. 2023 Oct 10;24(20):15046. doi: 10.3390/ijms242015046 (PMC10606003; doi:10.3390/ijms242015046)
Supplement: Supplementary file 1 [file ijms-24-15046-s001.zip › ijms-2645301-supplementary.pdf]

Supplementary

Table S1. Demographic, anthropometric and clinical features of the Liver Biopsy cohort (LBC, *n* = 1155).

	**LBC (*n* = 1155)**
Sex, M/F	546/609
Age, years	48.1 ± 12.8
BMI, kg/m^2	35.2 ± 8.6
IFG/T2D (0/1)	878/277
Total cholesterol, mmol/L	5.1 ± 1.07
LDL cholesterol, mmol/L	3.1 ± 0.97
HDL cholesterol, mmoL/L	1.3 ± 0.37
Triglycerides, mmoL/L	1.59 {0.98–1.91}
ALT, IU/l	32 {20–55}
AST, IU/l	24 {18–36}
GGT, IU/l	38 {21–78}
Steatosis ≥ 2, yes (%)	560 (48)
Lobular inflammation ≥ 1, yes (%)	749 (64)
Ballooning ≥ 1, yes (%)	333 (29)
Fibrosis ≥ 2, yes (%)	255 (22)
PLT count *10$^{\wedge 3}$	246 ± 71
FIB-4	1.22 ± 1.55
APRI	0.32 ± 0.42
Forns' index	4.46 ± 2.08
PNPLA3 rs738409, p.I148M	
CC, yes (%)	484 (41.9)
CG, yes (%)	485 (42)
GG, yes (%)	186 (16.1)

Values are reported as mean ± SD. number (%) or median {IQR}. as appropriate. BMI: body mass index; IFG: impaired fasting; T2D: type 2 diabetes mellitus.

Table S2. Association of the *PNPLA3* rs738409 C>G variant with hematological parameters and liver-related outcomes and in the UK Biobank cohort (UKBBC).

Phenotype	Estimate (β)	*p*-Value
K70: Alcoholic liver disease	0.0009	1.71×10^{-14}
K74: Fibrosis and cirrhosis of liver	0.0009	2.32×10^{-11}
K75: Other inflammatory liver diseases	0.0006	2.06×10^{-08}
K76: Other diseases of liver	0.002	2.51×10^{-22}
K70-K77: Diseases of liver	0.003	3.38×10^{-25}
I85 Oesophageal varices	0.0007	3.03×10^{-14}
Platelet count	-1.54	2.9×10^{-45}
Haemoglobin concentration	0.02	9.09×10^{-35}
Platelet crit	-0.001	3.62×10^{-29}
Haematocrit percentage	0.06	2.94×10^{-25}
Mean corpuscular haemoglobin	0.03	2.28×10^{-19}
Mean platelet (thrombocyte) volume	0.01	9.53×10^{-18}
Mean corpuscular volume	0.07	9.44e $\times 10^{-16}$
Monocyte percentage	0.04	4.70×10^{-15}
Mean sphered cell volume	0.07	1.15×10^{-10}
Red blood cell (erythrocyte) distribution width	-0.011	5.84×10^{-08}
Neutrophill count	-0.016	7.00×10^{-08}
White blood cell (leukocyte) count	-0.019	3.69×10^{-07}
Red blood cell (erythrocyte) count	0.003	1.97×10^{-06}
D65-D69 Coagulation defects, purpura and other haemorrhagic conditions	0.0005	0.018
D69 Purpura and other haemorrhagic conditions	0.0003	0.04

Biochemical parameters were assessed in the entire cohort (n = 500,000 subjects). HWE: 0.06; MAF: 0.22. Reference allele: G.

Table S3: Demographic, anthropometric and clinical features of 167 severely obese patients of whom RNAseq data were available.

	**Transcriptomic cohort (*n* = 167)**
Sex, M/F	28/139
Age, years	43±10
BMI, kg/m2	41.3±7.4
IFG/T2D (0/1)	150/17
Total cholesterol, mmol/L	5.3±1.2
LDL cholesterol, mmol/L	3.3±0.9
HDL cholesterol, mmoL/L	1.4±0.35
Triglycerides, mmoL/L	1.45±0.7
ALT, IU/l	16 {20-30}
AST, IU/l	15 {18-24}
GGT, IU/l	15 {24-43}
Steatosis ≥ 2, yes (%)	82 (49)
Lobular inflammation ≥ 1, yes (%)	94 (56)
Ballooning ≥ 1, yes (%)	24 (14)
Fibrosis ≥ 2, yes (%)	13 (8)
PLT count	274 ± 67
FIB-4	0.74 ± 0.48
APRI	0.20 ± 0.28
Forns' index	3.22 ± 1.47
PNPLA3 rs738409, p.I148M	
CC, yes (%)	78 (47)
CG, yes (%)	74 (44)
GG, yes (%)	15 (9)

Values are reported as mean ± SD. number (%) or median {IQR}. as appropriate. BMI: body mass index; IFG: impaired fasting; T2D: type 2 diabetes mellitus.

Table S4. Correlation analyses of gene expression of PNPLA3 and genes involved in PLT biosynthesis and clearance in *n* = 167 patients belonging to the Transcriptomic cohort.

mRNA	PNPLA3 expression	
	**Estimate (β)**	***p-Value***
DIAPH1	1.24	<0.0001
ETV6	0.09	<0.0001
IKZF5	0.16	<0.0001
MYH9	2.50	<0.0001
ORAI1	0.07	<0.0001
STIM1	0.40	<0.0001

Supplementary References

1. Dongiovanni, P., et al., *Transmembrane 6 superfamily member 2 gene variant disentangles nonalcoholic steatohepatitis from cardiovascular disease.* Hepatology, 2015. **61**(2): p. 506-14.
2. Meroni, M. and M. Longo, *The rs599839 A>G Variant Disentangles Cardiovascular Risk and Hepatocellular Carcinoma in NAFLD Patients.* 2021. **13**(8).
3. Meroni, M., et al., *Low Lipoprotein(a) Levels Predict Hepatic Fibrosis in Patients With Nonalcoholic Fatty Liver Disease.* 2022. **6**(3): p. 535-549.

Supplementary Figure Legends

Figure S1: Impact of the *PNPLA3* rs2294918 protective variant genotype on platelet count. Platelets distribution in 1155 MASLD patients, stratified according to the presence of the *PNPLA3* rs2294918 (p.E434K, G>A) variant (*n*=549 GG (48%); *n*=417 GA (36%) and *n*=189 AA (16%)). The estimates (β) were obtained from ordinal logistic regression analysis adjusted for gender, age, body mass index (BMI), type 2 diabetes (T2D) by using and additive (Model 1 °) or recessive model (Model 2 †) (**A**).

Figure S2: Impact of the *PNPLA3* genotype on non-invasive tests in predicting fibrosis stage. ROC curves describe the accuracy of the non-invasive scores, AST to Platelet Ratio Index (APRI) (**A**) and Forns' index (**B**) in discriminating the histological stage of fibrosis in 1155 MASLD patients stratified according to the presence of *PNPLA3* homozygosity (*n*=969 CC/CG and *n*=186 GG). Area under the curves (AUC) are reported in the graphs.

Figure S1

A

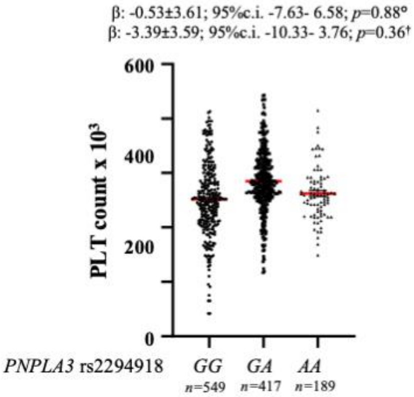

Figure S2

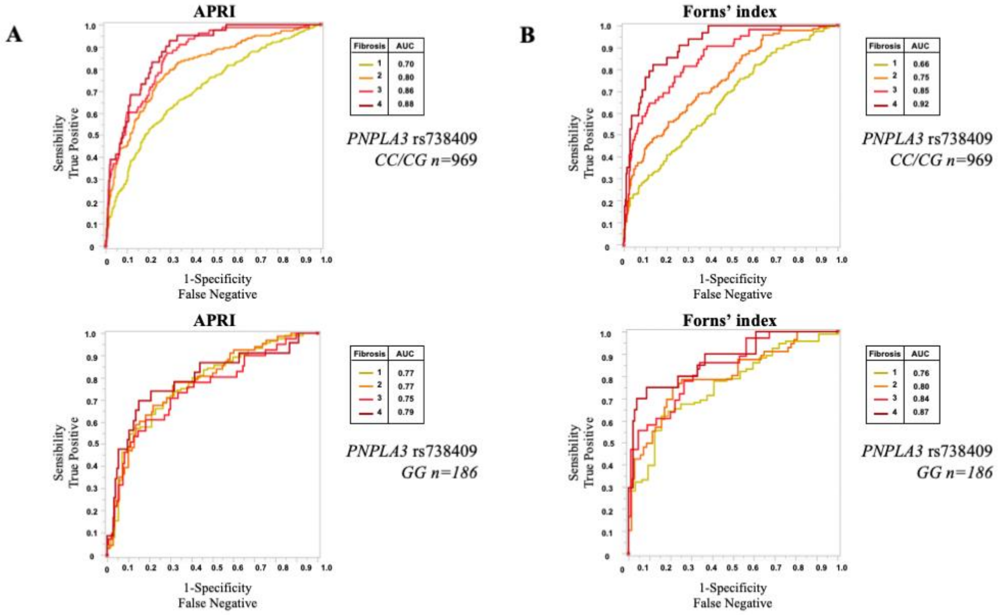

A
APRI
Sensibility
True Positive
1-Specificity
False Negative
Fibrosis AUC
1 0.70
2 0.80
3 0.86
4 0.88
PNPLA3 rs738409
CC/CG n=969
B
Forns' index
Fibrosis AUC
1 0.66
2 0.75
3 0.85
4 0.92
PNPLA3 rs738409
CC/CG n=969
APRI
Fibrosis AUC
1 0.77
2 0.77
3 0.75
4 0.79
PNPLA3 rs738409
GG n=186
Forns' index
Fibrosis AUC
1 0.76
2 0.80
3 0.84
4 0.87
PNPLA3 rs738409
GG n=186
